# Ethnic variation in asthma healthcare utilisation and exacerbation: systematic review and meta-analysis

**DOI:** 10.1183/23120541.00591-2022

**Published:** 2023-05-02

**Authors:** AbdulQadr Akin-Imran, Achint Bajpai, Dáire McCartan, Liam G. Heaney, Frank Kee, Charlene Redmond, John Busby

**Affiliations:** 1 Centre for Public Health, School of Medicine, Dentistry and Biomedical Sciences, Queen's University, Belfast, UK; 2 Faculty of Health and Life Sciences, Oxford Brookes University, Oxford, UK; 3 University of Central Lancashire, University of Central Lancashire Faculty of Clinical and Biomedical Sciences, Preston, UK; 4 Centre for Experimental Medicine, School of Medicine, Dentistry and Biomedical Sciences, Queen's University, Belfast, UK

## Abstract

**Background:**

Patients from ethnic minority groups (EMGs) frequently report poorer asthma outcomes; however, a broad synthesis summarising ethnic disparities is yet to be undertaken. What is the magnitude of ethnic disparities in asthma healthcare utilisation, exacerbations and mortality?

**Methods:**

MEDLINE, Embase and Web of Science databases were searched for studies reporting ethnic variation in asthma healthcare outcomes (primary care attendance, exacerbation, emergency department (ED) visits, hospitalisation, hospital readmission, ventilation/intubation and mortality) between White patients and those from EMGs. Estimates were displayed using forest plots and random-effects models were used to calculate pooled estimates. We conducted subgroup analyses to explore heterogeneity, including by specific ethnicity (Black, Hispanic, Asian and other).

**Results:**

65 studies, comprising 699 882 patients, were included. Most studies (92.3%) were conducted in the United States of America (USA). Patients from EMGs had evidence suggestive of lower levels of primary care attendance (OR 0.72, 95% CI 0.48–1.09), but substantially higher ED visits (OR 1.74, 95% CI 1.53–1.98), hospitalisations (OR 1.63, 95% CI 1.48–1.79) and ventilation/intubation (OR 2.67, 95% CI 1.65–4.31) when compared to White patients. In addition, we found evidence suggestive of increased hospital readmissions (OR 1.19, 95% CI 0.90–1.57) and exacerbation rates (OR 1.10, 95% CI 0.94–1.28) among EMGs. No eligible studies explored disparities in mortality. ED visits were much higher among Black and Hispanic patients, while Asian and other ethnicities had similar rates to White patients.

**Conclusions:**

EMGs had higher secondary care utilisation and exacerbations. Despite the global importance of this issue, the majority of studies were performed in the USA. Further research into the causes of these disparities, including whether these vary by specific ethnicity, is required to aid the design of effective interventions.

## Abstract 2

**Patients from ethnic minority groups have substantially increased secondary healthcare utilisation and exacerbations with evidence suggestive of lower primary care attendances #asthma** https://bit.ly/3lvKWgY

## Introduction

Asthma is one of the most common chronic diseases in the world, affecting >400 million patients [1]. Although it is prevalent across society [2], the burden of asthma is known to disproportionately affect patients from ethnic minority groups (EMGs). Compared with White Americans, EMGs have higher asthma prevalence, morbidity and adverse outcomes [3, 4]. Higher rates of emergency department (ED) visits, hospital admission and asthma mortality have been reported among EMGs when compared to White patients [3, 4].

Language barriers, poorer housing conditions and inadequate self-management have previously been considered as potential contributors to the observed ethnic disparities in asthma outcomes [5, 6]. Belonging to an EMG is associated with lower socioeconomic status in many countries [7], which can make it difficult to disentangle differences driven by ethnic factors such as culture, from those related to socioeconomic disadvantage. A recent study by the Severe Asthma Research Program in the USA found that the greater ED use observed in Black patients was entirely explained after accounting for socioeconomic circumstances and environmental factors [8]. Cultural differences in asthma medication adherence and health literacy [9, 10] have also been previously identified and are known to materially affect asthma outcomes [11, 12].

Despite substantial literature examining ethnic differences in asthma outcomes, previous systematic reviews have been limited to specific countries, populations (*e.g.* paediatrics) or outcomes [13, 14]. Despite a well-established literature on the topic from the USA, where issues around ethnic disadvantage are of particular interest, these studies have yet to be systematically synthesised. A broader analysis, including all relevant evidence worldwide, would help improve knowledge of potential ethnic inequalities and facilitate an investigation of where disparities are largest, and action is most needed to improve and standardise care.

## Methods

### Search strategy

A search strategy was developed to identify studies reporting differences in asthma healthcare outcomes and resource utilisation between White and EMG patients (supplementary tables S1 and S2). The specific outcomes of interest were primary care attendance, asthma exacerbation, ED visit, hospitalisation, ventilation/intubation, hospital readmission and asthma mortality. Three electronic databases (MEDLINE, Embase and Web of Science) were searched from inception (*i.e.* 1946 for MEDLINE, 1974 for Embase and 1997 for Web of Science) on 4 January 2023. Individual search results were combined, duplicates removed automatically using an online software (Covidence; Veritas Health Innovation, Australia) and manually checked. This systematic review was reported in accordance with the Preferred Reporting Items for Systematic Reviews and Meta-Analyses statement [15] and it was registered on PROSPERO prior to data extraction (registration number CRD42020200392). Patient representatives were not involved in the design, analysis or interpretation of the study.

### Study eligibility and selection

Studies were eligible if they were reported in peer-reviewed journals and all participants had physician-diagnosed or self-reported asthma. Asthma was defined as either a physician-diagnosed or self-reported (*e.g.* asthma diagnosis reported by the patient, but not corroborated through linkage to medical records). We excluded ecological studies as these are prone to the ecological bias [16]. Due to resource constraints our analysis was limited to studies published in the English language although a recent study concluded that this restriction is unlikely to have a material impact on the conclusion of a systematic review [17]. Titles and abstracts were screened independently by three reviewers (A. Akin-Imran, A. Bajpai and D. McCartan) and full texts were retrieved if potentially relevant. Three reviewers (A. Akin-Imran, A. Bajpai and D. McCartan) screened the full-text articles. Two independent reviewers (A. Akin-Imran and D. McCartan) conducted backward and forward citation searches on all studies initially included from the electronic search using Scopus (Elsevier, USA) and Web of Science (Clarivate Analytics, USA). Authors were not contacted to provide additional data outside that available in the published study. Throughout the process, disagreements were resolved by consensus or through referral to an additional reviewer (J. Busby).

### Data extraction and risk of assessment

Four reviewers (A. Akin-Imran, A. Bajpai, D. McCartan and C. Redmond) extracted data in duplicate using a pre-defined data extraction form (supplementary table S3). We extracted details on the study (country, design, clinical setting and time period), characteristics of the population (*e.g.* size, mean age, sex) and statistical methodology (*e.g.* confounder adjustment).

Odds ratios and their corresponding 95% confidence intervals comparing relevant outcomes were extracted. The principal quantitative synthesis involved a comparison of White patients *versus* those from EMGs. Univariable and multivariable effect estimates were retrieved; however, the multivariable estimate was used when possible. Unadjusted effect estimates and 95% confidence intervals were calculated manually where necessary using the methods outlined in the Cochrane Handbook [18]. Where studies presented multiple eligible estimates (*e.g.* separate estimates comparing Black *versus* White and Asian *versus* White), we used a within-study meta-analysis to calculate a pooled estimate comparing all EMGs *versus* White patients for that study.

The methodological quality of the included studies was assessed using the Newcastle–Ottawa Quality Assessment Scale for cohort, case–control [19] and cross-sectional [20] studies, using pre-defined assessment criteria (supplementary table S4). Studies were scored across three domains: selection of participants, comparability and ascertainment or assessment of the exposure and outcome. Domain scores were totalled, and each study was awarded an overall quality rating (poor or good).

### Data synthesis and analysis

Descriptive statistics including means, medians and percentages were calculated to summarise study characteristics. Meta-analyses were conducted using a random-effects model, as described by DerSimonian and Laird [21], with the degree of statistical heterogeneity assessed using the I^2^ statistic. Hazard ratios and relative risks were treated as odds ratios to facilitate the pooling of estimates. We conducted sensitivity analyses restricting to studies deemed good-quality. We undertook *s*everal pre-specified subgroup analyses to explore heterogeneity including by specific ethnic groups (Black, Hispanic, Asian and other), patient age (paediatric *versus* others), study data collection frame (pre- *versus* post-2010) and healthcare financing (public *versus* private). Subgroups were only compared if they comprised at least four studies, in line with recommendations [22]. Both funnel plots and Egger's test were used to investigate potential small study effects for all outcomes comprising ≥10 studies [23]. Analyses were performed using STATA (version 14).

## Results

**FIGURE 1:**
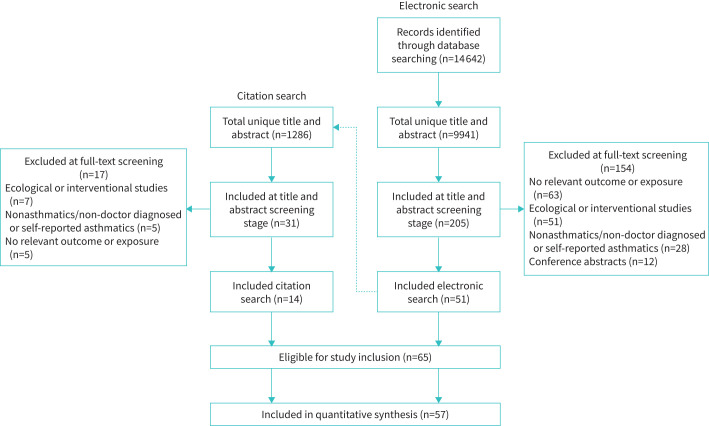
Preferred Reporting Items for Systematic Reviews and Meta-Analyses flowchart diagram outlining the study search and selection process.

The electronic search identified 9941 unique studies (figure 1), of which 205 were retained after title and abstract screening. A further 154 studies were excluded after full-text review due to failing to report a relevant outcome or exposure (n=63), an ecological study design (n=51), comprising nonasthmatic patients (n=28) or conference abstracts (n=12), leaving 51 eligible studies. An additional 14 studies were included following the forward and backward citation searches. Eight of the included studies were only included in the narrative synthesis: three studies [24–26] presented only narrative results, and five [27–31] did not provide sufficient details for comparative effect estimates to be derived. Therefore, 57 of the 65 studies were included in the meta-analysis.

### Quality assessment and small-study effects

The methodological quality of the included studies was deemed good in 37 (56.9%) studies (supplementary table S5). The most common factor for a study being deemed poor-quality was a failure to adjust for at least age and/or sex, which occurred in all but three of the 28 poor-quality studies. Within the cohort studies, there were also potential issues with outcome ascertainment resulting from patient self-report (n=8, 38.1% of poor-quality studies), whereas in cross-sectional studies the response rate was frequently unsatisfactory (n=12, 60.0%). Overall, studies differed considerably in the level of statistical adjustment used in their analysis. Of those that performed some adjustment (n=39), most (89.7%) adjusted for age and/or sex, while some additionally accounted for income (41.0%), asthma severity (33.3%) and education (30.8%) (supplementary table S6).

There was some evidence of small-study effects in studies investigating ED visits (p<0.001), with the funnel plot displaying a marked asymmetry in which substantially more studies reported marked ethnic disparities than would have been expected (supplementary figure S1). However, no indication of small­-study effects was observed for hospitalisations (supplementary figure S2).

### Study characteristics

The 65 included studies included 699 882 patients, with the population size varying considerably across studies, from 80 to 133 509 patients. Most studies (n=35, 53.8%) were published before 2010 and the majority were conducted in the USA (n=60, 92.3%), including three set in both the USA and Canada [32–34]. Three studies [35–37] were conducted in the United Kingdom (UK), while a single study was conducted in each of New Zealand [38] and Canada [39] (table 1). The majority of studies were cohort (n=44, 67.7%) or cross-sectional (n=20, 30.8%), with only one employing a case–control design. The median (interquartile range) age of study populations was 35.2 (10.1–49.5) years, with 23 (43.1%) studies reporting solely on paediatric patients. Most studies investigated the effect of ethnicity on multiple outcomes, but most commonly reported on differences in ED visits (n=41, 40.6%) and hospitalisations (n=33, 32.7%). Studies rarely investigated differences in exacerbation (n=10, 9.9%), hospital readmission (n=6, 5.9%), ventilation/intubation (n=5, 5.0%) and primary care attendance (n=4, 4.0%). None of the studies included in our review reported on asthma mortality (table 1).

**TABLE 1:** Demographic and characteristics of studies evaluating healthcare utilisation of asthmatic patients

**First author, year [reference]**	**Country**	**Design**	**Patients**	**Female, %**	**Mean age, years**	**Clinical setting**	**Exposure**	**Outcomes measured**
**Mitchell, 1988 [38]**	New Zealand	CS	355	NS	5.8	General population^#^	Asian	Readmission
**Lozano, 1995 [40]**	USA	CS	1945	41.7	8.1	Insurance	Black	ED visit, hospitalisation, PCA
**Sarpong, 1997 [41]**	USA	CS	138	65.0	10.1	Secondary care	Black	Hospitalisation
**Joseph, 1998 [42]**	USA	CS	570	38.0	NS	Secondary care	Black	ED visit, hospitalisation
**Zoratti, 1998 [25]**	USA	CSS	2073	65.0	30.7	Insurance	Black	ED visit, hospitalisation, PCA
**Blixen, 1999 [43]**	USA	CS	193	77.2	37.3	Hospitalisation record	Black	ED visit, PCA
**Meurer, 2000 [44]**	USA	CSS	307	NS	NS	General population^#^	Black, Hispanic	ED visit
**Eisner, 2001 [45]**	USA	CS	242	73.0	40.5	General population^#^	Black, Hispanic, Asian	Hospitalisation
**Krishnan, 2001 [46]**	USA	CSS	5062	71.6	44.0	General population^#^	Black	ED visit, hospitalisation
**Ortega, 2001 [47]**	USA	CS	1002	37.0	5.3	General population^#^	Black, Hispanic	ED visit, hospitalisation
**Ortega, 2001 [48]**	USA	CS	897	37.3	NS	General population^#^	Black, Hispanic	ED visit, hospitalisation
**Amre, 2002 [39]**	Canada	CS	457	44.1	NS	ED record	Other	ED visit, hospitalisation
**Diette, 2002 [49]**	USA	CS	6590	70.1	NS	General population^#^	Black, other	Hospitalisation
**Lafata, 2002 [50]**	USA	CS	452	37.0	8.7	Insurance	Black, other	ED visit, hospitalisation
**Weber, 2002 [32]**	USA and Canada	CS	1805	65.3	35	ED record	Black, Hispanic	Hospitalisation
**Bloomberg, 2003 [33]**	USA	CS	8761	37.2	NS	Hospitalisation record	Black, other	Readmission
**Boudreaux, 2003 [34]**	USA and Canada	CS	1800	65.2	34.7	ED record	Black, Hispanic	ED visit, hospitalisation
**Boudreaux, 2003 [51]**	USA and Canada	CS	1095	40.2	7.8	ED record	Black, Hispanic	Hospitalisation, intubation
**Shields, 2004 [52]**	USA	CS	5773	40.6	NS	Insurance	Black	ED visit, hospitalisation
**Carroll, 2005 [53]**	USA	CS	4315	100	NS	Insurance	Black	ED visit, hospitalisation
**Grant, 2005 [54]**	USA	CSS	152	61.8	NS	General population^#^	Black	ED visit, hospitalisation
**Griswold, 2005 [55]**	USA	CS	3151	63.1	35.2	General population^#^	Black, Hispanic, other	ED visit
**Ash, 2006 [56]**	USA	CS	10 145	67.4	NS	Hospitalisation record	Black, Hispanic, other	Readmission
**Meng, 2006 [57]**	USA	CSS	4359	68.2	NS	General population^#^	Black, Hispanic, Asian, other	ED visit
**DeWalt, 2007 [58]**	USA	CSS	150	NS	7.7	General population^#^	Black	ED visit, hospitalisation
**Erickson, 2007 [59]**	USA	CS	678	71.2	61.1	Hospitalisation record	Black	ED visit, hospitalisation
**Forester, 2008 [60]**	USA	CSS	80	45.0	9	General population^#^	Black, Hispanic	Hospitalisation
**Haselkorn, 2008 [61]**	USA	CS	2128	71.9	50.3	General population^#^	Black	ED visit
**Chandra, 2009 [62]**	USA	CS	1232	55.7	NS	Hospitalisation record	Black, Hispanic	Hospitalisation
**Crocker, 2009 [63]**	USA	CSS	1485	NS	NS	General population^#^	Black, Hispanic	ED visit, hospitalisation
**Diette, 2009 [64]**	USA	CSS	406	42.3	10.2	General population^#^	Black, other	Hospitalisation, PCA
**Gorman, 2009 [65]**	USA	CSS	133 509	51.7	45.2	General population^#^	Black, Hispanic, Asian, other	ED visit
**Haselkorn, 2009 [66]**	USA	CS	563	31.3	NS	General population^#^	other	Exacerbation
**Kim, 2009 [67]**	USA	CSS	982	36.2	NS	General population^#^	Black, Hispanic	ED visit
**Wright, 2009 [68]**	USA	CSS	1313	38.0	NS	General population^#^	Black, Hispanic	ED visit
**Carroll, 2010 [69]**	USA	CS	306	42.2	8.1	Hospitalisation record	Black, Hispanic	Exacerbation
**Canino, 2012 [70]**	USA	CSS	804	43.6	10.6	General population^#^	Hispanic	ED visit
**Hasegawa, 2014 [71]**	USA	CS	86 224	NS	NS	Insurance	Black, Hispanic, other	ED visit
**Kenyon, 2014 [72]**	USA	CS	36 601	38.9	NS	Hospitalisation record	Black, Hispanic, other	Readmission
**Lee, 2014 [73]**	USA	CSS	1323	67.2	74.4	General population^#^	Black, Hispanic, Asian, other	ED visit
**Auger, 2015 [74]**	USA	CS	601	NS	5.1	Tertiary care	Black, other	Readmission
**Venkat, 2015 [75]**	USA	CS	1785	59.8	NS	Secondary care	Black, Hispanic	ED visit, hospitalisation
**Wells, 2015 [76]**	USA	CCS	719	74.1	32.4	Secondary care	Black, Hispanic, other	ED visit
**Hull, 2016 [35]**	UK	CS	35 864	53.3	NS	Primary care	Black, Asian	Hospitalisation
**Mitchell, 2016 [26]**	USA	CS	273	41.4	7.5	General population^#^	Black	Hospitalisation, intubation
**Franklin, 2017 [77]**	USA	CS	265	42.6	11.5	Hospitalisation record	Black	ED visit, hospitalisation, intubation
**Hughes, 2017 [78]**	USA	CSS	33 201	49.7	NS	General population^#^	Black, Hispanic, Asian	ED visit
**Parikh, 2017 [79]**	USA	CS	36 906	37.4	5	Tertiary care	Black, Hispanic, other	Readmission
**Zhang, 2017 [80]**	USA	CSS	5535	42.7	11.8	General population^#^	Black, Hispanic	ED visit
**Deshpande, 2018 [24]**	USA	CSS	5672	68.3	49.3	General population^#^	Black, Hispanic	ED visit
**Grunwell, 2018 [81]**	USA	CS	579	41.8	NS	Secondary care	Black, other	Hospitalisation, intubation
**Trent, 2018 [82]**	USA	CS	913	53.3	NS	Hospitalisation record	Black, Hispanic	ED visit, hospitalisation, intubation
**Aratani, 2020 [83]**	USA	CS	47 657	36.0	2.7	Secondary care	Black, Hispanic, Asian, other	Hospitalisation
**Fitzpatrick, 2019 [8]**	USA	CS	631	59.3	38.2	Secondary care	Black	ED visit, hospitalisation
**Cremer, 2020 [84]**	USA	CSS	4700	67.3	66.5	General population^#^	Black, Hispanic	ED visit, hospitalisation
**Urquhart, 2020 [85]**	USA	CSS	3336	41.3	NS	General population^#^	Black, Hispanic	ED visit
**Zein, 2021 [86]**	USA	CS	60 302	62.1	47.5	Secondary care	Black, Asian, other	Exacerbation
**Banta, 2021 [87]**	USA	CSS	61 625	49	NS	General population^#^	Hispanic, other	ED visit, exacerbation
**Kraft, 2021 [27]**	USA	CS	5701	66	49.5	Hospitalisation record	Black, Asian, other	Exacerbation
**Sheikh, 2021 [30]**	USA	CS	345	44.6	6.2	Tertiary care	Black	ED visit
**Adejare, 2022 [29]**	USA	CS	42 375	67.2	49.6	Hospitalisation record	Black	ED visit
**Beuther, 2022 [31]**	USA	CS	1112	70.5	43.9	Tertiary care	Other	Exacerbation
**Busby, 2022 [37]**	UK	CS	3402	63.6	50.0	Tertiary care	Black, Asian, other	ED visit, exacerbation
**Busby, 2022 [37]**	UK	CS	13 936	67.9	55.8	Primary care	Black, Asian, other	ED visit, exacerbation
**Lugogo, 2022 [28]**	USA	CS	1884	69	54	Tertiary care	Black, Hispanic, other	ED visit, hospitalisation, exacerbation
**Redmond, 2022 [36]**	UK	CS	1140	61.1	50.6	Hospitalisation record	Other	Exacerbation

Data are presented as n, unless otherwise stated. CS: cohort study; NS: not specified; ED: emergency department; PCA: primary care attendance; CSS: cross-sectional study; CCS: case–control study. ^#^: survey/interview.

### The association between ethnicity and healthcare utilisation

**FIGURE 2:**
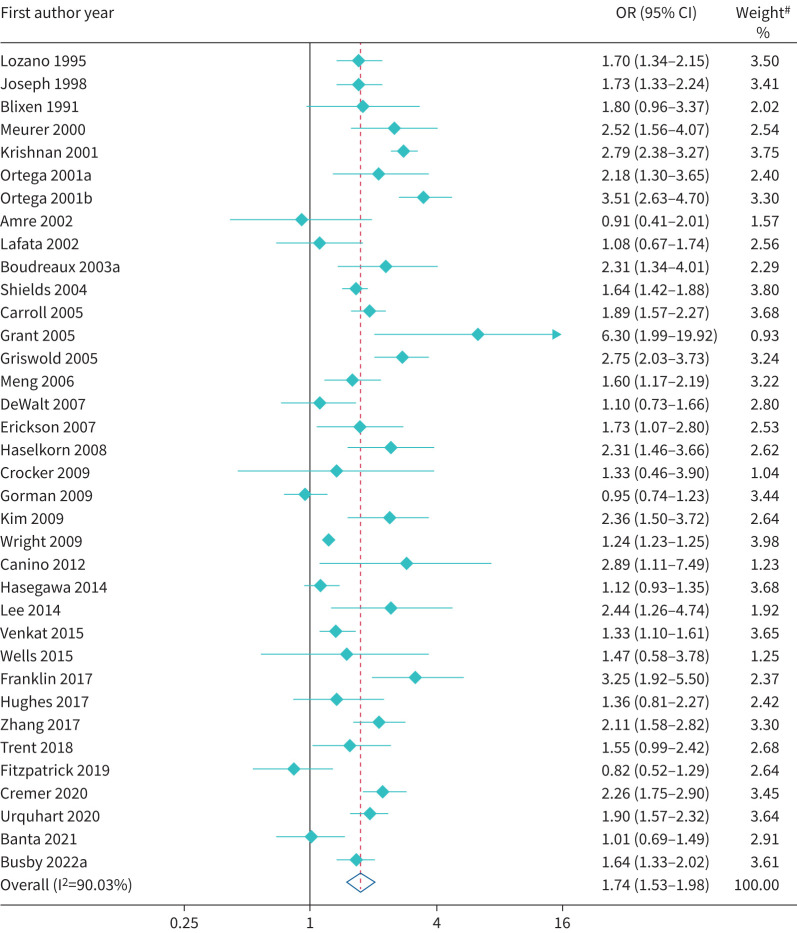
Forest plot of odds ratio of asthma-related emergency department visits. ^#^: weights are from random-effects model.

**FIGURE 3:**
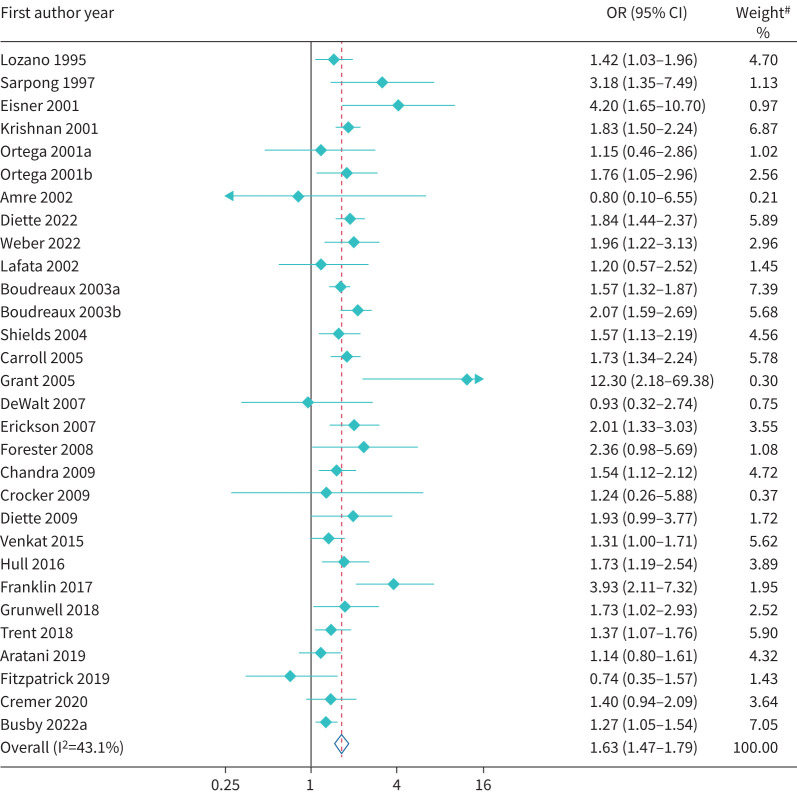
Forest plot of odds ratio of asthma-related hospitalisation. ^#^: weights are from random-effects model.

Primary care attendances were lower among EMG patients when compared to White patients; however, this was imprecisely estimated (OR 0.72, 95% CI 0.48–1.09; I^2^=70.5%) (table 2 and supplementary figure S3) compared to White patients. However, EMGs had higher secondary healthcare utilisation (ED visits, hospital admission and hospital readmission). The proportion attending ED was higher (OR 1.74, 95% CI 1.53–1.98) among patients of EMGs than their White counterparts, although there was evidence of between-study heterogeneity (I^2^=90.3%) (figure 2). Findings of higher ED visits among EMGs were largely consistent across individual studies (91.7%), including within a recent study from the USA which reported that 14% of White patients attended the ED in the past 12 months due to their asthma compared to 32% of Black patients (adjusted OR 2.34, 95% CI 1.65–3.33; p<0.001) and 23% of Hispanic patients (adjusted OR 2.17, 95% CI 1.51–3.10; p<0.001) [84]. Similarly, patients from EMGs were substantially more likely to be hospitalised (OR 1.63, 95% CI 1.48–1.79; I^2^=43.1%) (figure 3), while there was a trend for increased rates of hospital readmissions (OR 1.19, 95% CI 0.90–1.57; I^2^=78.0%) (supplementary figure S4). In addition, patients from EMGs were more likely to be ventilated/intubated (OR 2.67, 95% CI 1.65–4.31; I^2^=74.2%) (supplementary figure S5). The magnitude of these differences varied between studies and there was substantial heterogeneity between studies across all outcomes.

**TABLE 2:** Pooled effect of ethnicity on asthma-related healthcare utilisation and morbidity

	**All studies**			**Good-quality studies**		
	**Studies**	**OR^#^ (95% CI)**	**I^2^**	**Studies**	**OR^#^ (95% CI)**	**I^2^**
**Healthcare utilisation**						
Primary care attendance	4	0.72 (0.48–1.09)	70.5	3	0.61 (0.44–0.83)	6.9
ED visit	36	1.74 (1.53–1.98)	90.3	21	1.74 (1.56–1.93)	49.8
Hospitalisation	30	1.63 (1.48–1.79)	43.1	15	1.54 (1.35–1.77)	29.9
Hospital readmission	6	1.19 (0.90–1.57)	78.0	5	1.12 (0.84–1.51)	79.3
Ventilation/intubation	5	2.67 (1.65–4.31)	74.2		NS	
**Exacerbations**	7	1.10 (0.94–1.28)	78.8	5	1.06 (0.91–1.23)	78.6

Data are presented as n, unless otherwise stated. ED: emergency department; NS: not specified. **^#^**: ethnic minority groups *versus* White patients.

### The association between ethnicity and asthma exacerbations

Few studies explored the association between ethnicity and asthma exacerbation (table 2), with most (71.4%) reporting higher rates among EMGs (pooled OR 1.10, 95% CI 0.94–1.28; I^2^=78.8%) (supplementary figure S6).

### Sensitivity and subgroup analyses

Our overall conclusions were similar in sensitivity analyses restricted to studies deemed good-quality; however, we found a stronger and statistically significant relationship between EMGs and lower primary care attendance in this analysis (OR 0.61, 95% CI 0.44–0.83; I^2^=6.9%) (table 2). The pooled estimate for the other outcomes were similar when restricting to good quality studies, and in most cases, the heterogeneity was substantially reduced (table 2).

The pattern of secondary healthcare utilisation differed by specific ethnicity (supplementary table S7). Rates of ED visits were much higher among Black (OR 1.86, 95% CI 1.68–2.06) and Hispanic (OR 1.52, 95% CI 1.35–1.71) patients (p=0.001), while Asian (OR 0.97, 95% CI 0.59–1.61) and those from other ethnicities (OR 1.13, 95% CI 0.86–1.48) had similar rates to White patients. Black patients also had higher rates of hospitalisation (OR 1.69, 95% CI 1.40–2.06) than Hispanic patients (OR 1.33, 95% CI 1.09–1.62), Asian patients (1.42, 95% CI 0.85–2.38) or those from other ethnicities (OR 1.23, 95% CI 0.88–1.72) (p=0.068), and higher rates of readmission (OR 1.76, 95% CI 1.61–1.91) than those from other ethnicities (OR 1.05, 95% CI 0.97–1.13) (p<0.001). There was little evidence of any difference in disparities by age (ED visits and hospitalisations) or time period (ED visits) (supplementary table S7). Data were not available to allow robust subgroup analyses for other outcomes, and no subgroup analyses comparing healthcare system funding models were possible.

## Discussion

In this systematic review, comprising 65 studies and 699 882 asthma patients, we found that patients from EMGs had higher secondary healthcare utilisation, evidence to suggest increased rates of exacerbation, and, when restricting to good-quality studies, reduced primary care attendance when compared to White patients. Black and Hispanic patients had particularly high rates of secondary healthcare utilisation, while rates among those from Asian and other ethnicities were relatively similar to White patients. Despite the global importance of this issue, the vast majority of relevant research was conducted in the USA, and most eligible studies were published ≥10 years ago.

Our findings are consistent with other systematic reviews in asthma [13, 14], which have reported poorer healthcare outcomes among EMGs. Importantly, these reviews were limited to only UK-based studies (published >15 years ago) [13], or paediatric patients [14], while the current review provides an update of this evidence and includes all relevant studies worldwide regardless of patient demographics. The findings in this review are consistent with findings of ethnic disparities across a broad array of diseases and outcomes [88–91]. Compared to White patients, Black patients had greater COPD severity [88, 89] and were more likely to be admitted to hospital for respiratory causes [89]. Additionally, increased hospitalisation and mortality have been observed among EMGs in coronary health disease, congestive heart failure and cerebrovascular disease [90, 91].

Minority ethnicity has been related to increased obesity and comorbidities, which are known correlates of asthma morbidity [92], and is strongly associated with lower socioeconomic status in many countries [7]. Evidence from the Severe Asthma Research Program in the USA has found that socioeconomic factors are a strong mediator of ethnic disparities in asthma ED attendance [8]. This could be particularly relevant for the results of our review, as the studies included were overwhelmingly based in the USA, where issues relating to variable healthcare insurance and poverty are common among EMGs, and in particular Black and Hispanic patients [93, 94]. We did not find any evidence of higher rates of ED attendance among Asian patients, who have a more similar socioeconomic status to the White population within the USA [94].

EMGs, particularly those with limited English proficiency living in countries where this is the primary language [95], and limited reading ability [10], are known to have lower health literacy [11]. This is important, as poor health literacy has been associated with incorrect metered-dose inhaler technique [10], increased likelihood of inpatient visits [95, 96], emergency care [96] and worse asthma control [95]. EMGs express greater concern regarding preventive asthma medications [97] and are known to have poorer adherence to their maintenance asthma medications, which has been consistently associated with poorer outcomes in several studies [12]. Inadequate living conditions among EMGs may also contribute to the development and severity of asthma symptoms, and reduced asthma control, possibly due to increased allergen exposure [14].

When restricting to good-quality studies we found evidence of lower primary care attendances, but higher secondary healthcare utilisation among the EMGs, which may reflect differences in healthcare-seeking behaviour [13, 98]. The decision to seek primary or secondary care interventions are influenced by various factors, including those related to socioeconomic circumstances and specialist accessibility [99]. One USA-based study reported significantly fewer primary care attendances among African Americans when compared to Whites even within the same insurance scheme, suggesting that cultural factors and personal beliefs may also be important contributory factors [43]. Substantial evidence has shown that African American patients are more reliant on ED services for asthma and other conditions [100, 101]. Similarly, studies have demonstrated poor awareness of the need for preventative care among Black men, who often wait until symptoms appear to seek treatment, and sometimes prioritise treatment for their family rather than themselves [102].

There is some evidence that EMGs have poorer satisfaction with their healthcare providers and have been reported to receive poorer care with lower levels of adherence to guidelines and a greater propensity to underestimate asthma severity than their White counterparts [103–105]. Children from minority backgrounds are less likely to have their asthma treatment escalated and use more oral corticosteroid at all treatment steps [106–108]. In addition, racism and mistrust of healthcare providers have been shown to result in fewer visits to primary healthcare centres and an increased tendency to attend the ED, which could have contributed to our results [102]. It should be noted that many of the other potential drivers of poorer outcomes among EMGs cited previously, including lower socioeconomic status and poorer housing conditions, may also be driven by racism or structural inequality as a root cause [7].

Given the myriad factors that are potentially driving ethnic disparities, solutions are unlikely to be straightforward. Evidence suggests that culturally sensitive and tailored interventions are required to mitigate asthma ethnic disparities [109]. A recently updated Cochrane review demonstrated the benefits of culturally specific asthma education programmes in reducing severe asthma exacerbations in minority children, compared to generic programmes or usual care [109]. A citywide asthma management programme directed towards poor, minority, urban children in the USA found that adherence to anti-inflammatory guidelines by primary care providers reduced asthma-related hospitalisations, ED visits and outpatient visits [110]. Interventions to improve medication adherence and health literacy are typically cited as potential techniques to reduce health inequalities; however, more work is needed to evaluate their cost-effectiveness and explore their impact on healthcare outcomes [111, 112]. A recent review of patient and family-led interventions aiming to improve inhaled corticosteroid adherence among Black/African Americans reported that no randomised controlled trials have found a statistically significant improvement in adherence [113].

This review is novel, as it is the first to systematically report on the association of ethnicity on various aspects of asthma healthcare outcomes and it was not restricted to specific countries or patient demographics. Our results provide an at-a-glance summary of all relevant studies and our exploration of heterogeneity highlights specific subgroups where disparities are largest and action is urgently needed to standardise care.

Several of the studies included in our primary analysis were of poor quality and there were high levels of heterogeneity for several outcomes, which could be related to differences in outcome classification, exposure definition, methodology (*e.g.* type of effect estimate) or study definition, and may hinder the interpretability of our results. However, this was largely reduced when restricted to good-quality studies and our overall conclusions of increased secondary healthcare utilisation and exacerbations among EMGs were unaltered. Although we used subgroup analyses to explore heterogeneity, this was limited by the analyses that were reported in the primary studies. Data on important subgroups, for example sex-specific estimates, were generally not available. We restricted our review to studies that used White patients as their reference group, which may have biased our analysis to Northern American and European countries.

Our primary analysis presented all EMGs combined; however, this is unlikely to be valid. Although we presented separate estimates for Black, Hispanic and Asian patients, important differences are likely to exist even within these groups. For example, Mexican Americans have markedly lower asthma morbidity than Puerto Ricans [4]. The majority of the studies included in this review were based in the USA, which may limit the generalisability of our results. We believe that this is an important finding in itself and may suggest a lack of research into the magnitude and drivers of ethnic disparities in asthma outcomes within many countries. Despite our finding of a broad literature on ethnic disparities among adults living in the USA, this evidence had not previously been considered within a systematic review prior to the present study. Consequently, our results provide new and important evidence for practitioners in that region. Surprisingly, no studies have explored ethnic differences in mortality using individual patient data and this is an important area for future research. Lastly, our study did not include data on intermediate outcomes such as asthma control.

### Conclusion

In summary, EMGs with asthma had substantially higher rates of secondary healthcare utilisation and exacerbations with evidence suggestive of lower primary care attendances. Despite the global importance of this issue, the vast majority of relevant studies were from the USA and it remains largely unclear whether these disparities are replicated in other countries with different populations and healthcare funding models. Further research into the causes of these disparities, including whether these vary by specific ethnicity, is required to aid the design of effective interventions. To understand the mechanisms behind these disparities it is likely that innovative ways of combining qualitative and quantitative studies and of examining mediation effects will need to be pursued or developed further [114–116].

## Supplementary material

10.1183/23120541.00591-2022.Supp1 **Please note:** supplementary material is not edited by the Editorial Office, and is uploaded as it has been supplied by the author.
Supplementary figures 00591-2022.supplement_figures
Supplementary tables 00591-2022.supplement_tables
