# Supplementary material for: Ethnic variation in asthma healthcare utilisation and exacerbation: systematic review and meta-analysis
Source: ERJ Open Res. 2023 May 2;9(3):00591-2022. doi: 10.1183/23120541.00591-2022 (PMC10152257; doi:10.1183/23120541.00591-2022)
Supplement: Supplementary file 1 [file 00591-2022.supplement_figures.pdf]

Online Data Supplement

Title: Variation in asthma care, exacerbations and mortality by ethnicity: A systematic review and meta-analysis.

Authors: AbdulQadr Akin-Imran, PhD[1,2], Achint Bajpai, BSc[3], Dáire McCartan[1], Liam G Heaney, MD[4], Frank Kee, MD[1], Charlene Redmond, BSc[1], John Busby, PhD[1]

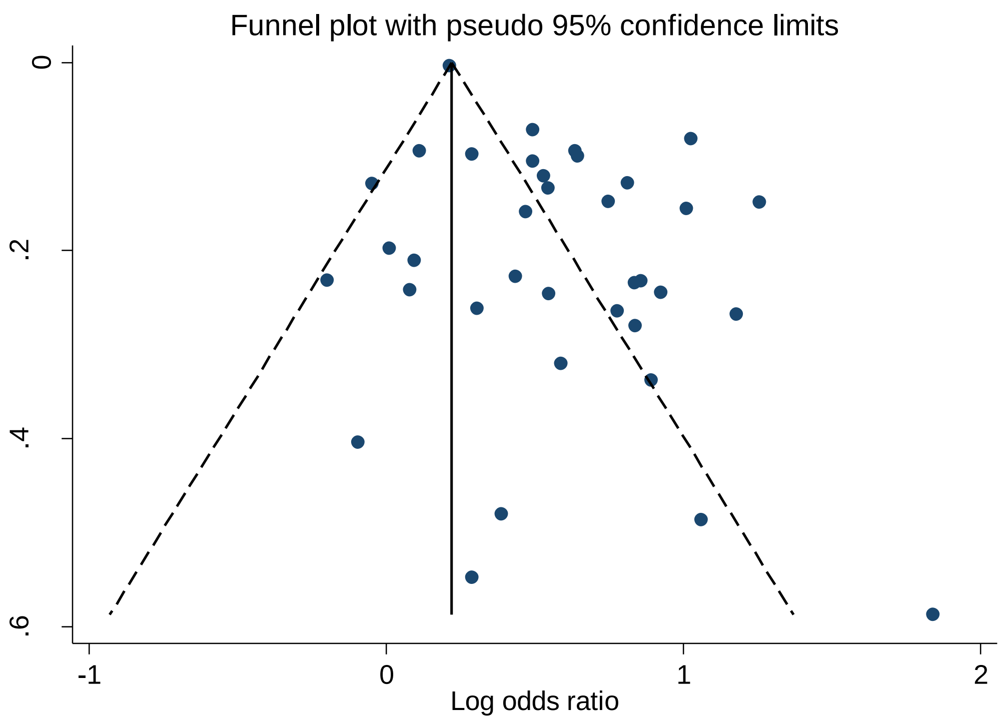

Figure S1: Funnel plot for studies reporting emergency department visits by different ethnic minority groups

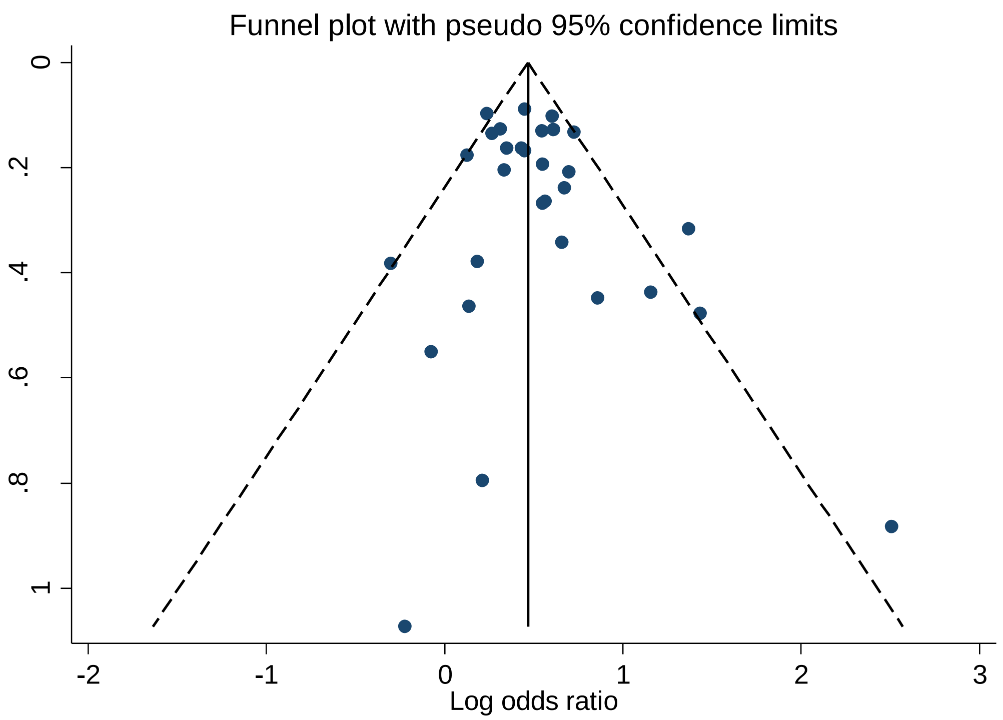

Figure S2: Funnel plot for studies reporting hospitalisations by different ethnic minority groups

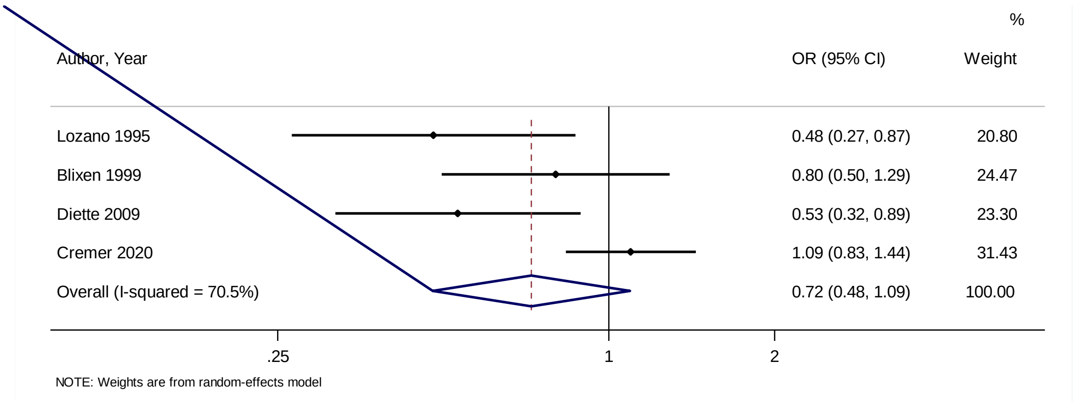

Figure S3: Forest plot of odds ratio of asthma-related primary care attendance

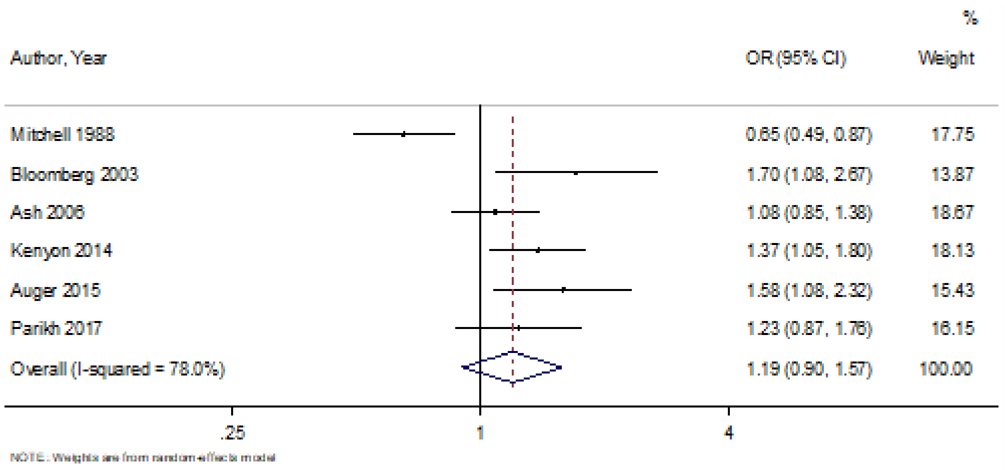

Figure S4: Forest plot of odds ratio of asthma-related hospital readmission

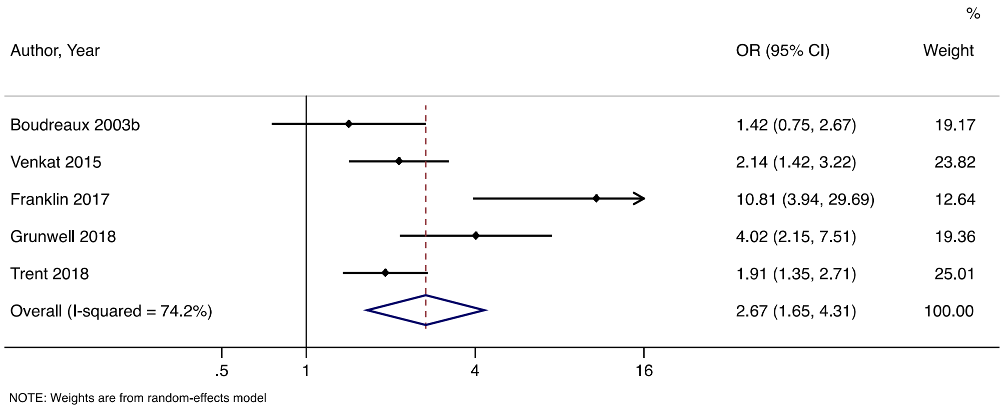

Figure S5: Forest plot of odds ratio of asthma-related ventilation / intubation

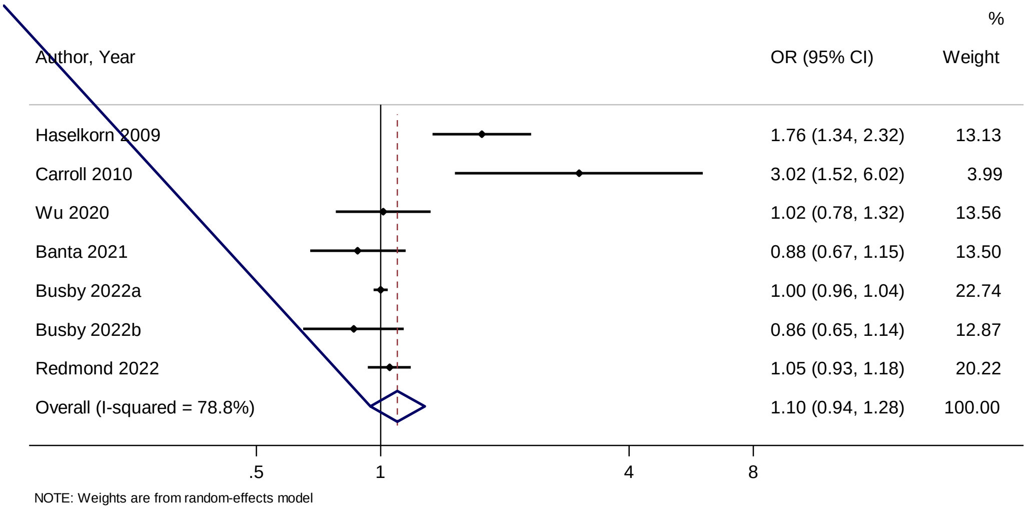

Figure S6: Forest plot of odds ratio of asthma-related exacerbation
