# Supplementary material for: Ethnic variation in asthma healthcare utilisation and exacerbation: systematic review and meta-analysis
Source: ERJ Open Res. 2023 May 2;9(3):00591-2022. doi: 10.1183/23120541.00591-2022 (PMC10152257; doi:10.1183/23120541.00591-2022)
Supplement: Supplementary file 2 [file 00591-2022.supplement_tables.pdf]

Online Data Supplement

Title: Variation in healthcare utilisation, exacerbations and mortality by ethnicity: A systematic review and meta-analysis.

Authors: AbdulQadr Akin-Imran, PhD[1,2], Achint Bajpai, BSc (Hons)[3], Dáire McCartan[1], Liam G Heaney, MD[4], Frank Kee, MD[1], Charlene Redmond, BSc[1], John Busby, PhD[1]

Table S1: Search domains and terms used in MEDLINE and EMBASE database

MEDLINE and EMBASE search strategy: Ovid <inception to July 2020>

Domain	Search Terms
**Asthma**	Asthma/ or asthma.mp. OR wheeze.mp. **AND**
**Ethnic/race**	Ethnic Groups/ or ethnic*.mp. OR race*.mp. OR racial group.mp. OR migration.mp. OR emigrant*.mp. OR migrant*.mp. OR "Emigrants and Immigrants"/ or "Emigration and Immigration"/ OR Minority Groups/ or minorit*.mp. OR Minority Health/ OR Refugees/ or refug*.mp. OR Refugee Camps/ OR asylum.mp. OR Indian.mp. OR Pakistani.mp. OR Bangladeshi.mp. OR Asian.mp. OR Black.mp. OR Afro*.mp. OR African Americans/ OR African.mp. OR Chinese.mp. OR BAME.mp. OR Eastern Europe.mp. OR Europe, Eastern/ OR Eastern Europe*.mp. OR Europe, Eastern/ OR Irish.mp. OR Non?White*.mp. OR Arabs.mp. or Arabs/ OR Gyps*.mp. OR Gips*.mp. OR Jews/ OR Jew*.mp. OR Hispanic Americans/ OR Hispanic*.mp. OR Latin America/ or Latin*.mp. OR white.mp. OR mixed.mp. OR Caribbean.mp. OR American Indian.mp. or Indians, North American/ OR Alaska Native.mp. or Alaska Natives/ OR Native Hawaiian.mp. OR Pacific Islander.mp. **AND**
**Outcome**	Mortality/ or mortality.mp. OR Death/ or death*.mp. OR morbidity.mp. or Morbidity/ OR exacerbat*.mp. OR vent*.mp. OR emergency department.mp. or Emergency Service, Hospital/ OR ED.mp. OR A&E.mp. OR (accident and emergency).mp. OR accident & emergency.mp. OR emergency room.mp. OR emergency ward.mp. OR Patient Readmission/ or readmission*.mp. OR hospitali?ation.mp. OR Patient Admission/ or admission*.mp. OR General Practice/ or general pract*.mp. or Primary Health Care/ or General Practitioners/ OR primary care.mp. OR attend*.mp. OR consult*.mp. OR utili*.mp. OR Intensive Care Units/ or ICU.mp. OR (High Dependency Unit* or HDU).mp. OR critical care.mp. or Critical Care/

Table S2: Search domains and terms used in Web of Science database

Web of Science search strategy: <Inception to July 2020>

Domain	Search Terms
**Asthma**	TS= (asthma OR wheeze) **AND**
**Ethnic/race**	TS= (ethnic* OR ethnic groups OR race* OR racial group OR migration OR migrant* OR minorit* OR minority group OR minority health OR emigrant* OR immigrant* OR immigrant OR refug* refugee camp OR asylum OR Indian OR Pakistani OR Bangladeshi OR Asian OR Black OR Afro* OR African OR African American OR Chinese OR BAME OR Eastern Europe* Irish OR Non white* OR arabs/ or gypsies/ or jews* OR Hispanic* OR Latin* OR Gyps* OR Gips*) **AND**
**Outcome**	TS= (mortality OR death* OR morbidity OR exacerbat* OR vent* OR “emergency department” OR ED OR (accident near/2 emergency) OR A&E OR readmission* OR hospitali?ation OR admission* OR “primary care” OR GP OR “general pract*” OR *attend* or consult* or utili*)

Table S3: List of data fields collected during data extraction

Study details	Categorical variable options
Title	
Lead author contact details	
Cohort description	
Country in which the study was conducted	
Study design	Case control, cohort, cross-sectional
Data collection setting	General practice, hospitalization records, ED records, secondary care, tertiary care, insurance database, general population, other
Study year / Data collection period	
Total number of participants	
Mean Age (SD/SE)	
Female (%)	
Age range	
Inhaled Corticosteroid (%)	
Oral Corticosteroid (%)	
Severe asthma (%)	
Asthma severity	Not specified, mild, moderate, severe, other
FEV1 Average	

Study comparison data	Categorical variable options / examples
Exposure	Race, Ethnicity, Race/Ethnicity
Group compared	e.g., White vs Hispanic
Reference group summary	i.e., White
Estimate group summary	e.g., Black
How outcomes were compared	e.g., the probability of ED visit (odds ratio) or counting the number of admissions (rate ratio)
Outcomes	primary care attendance, exacerbations, ED visit, hospitalisation, ventilation / intubation, readmission, Mortality
Ratio type	Odds ratio, risk ratio, hazard ratio, chi squared
Ratio	
Lower CI	
Upper CI	
Confidence level (%)	
P value	
Standard error	
Are estimates adjusted?	Yes, no
Variables adjusted for	
Were estimates calculated manually?	Yes, no
Outline how estimate was calculated	

Table S4: Quality assessment criteria, based on Newcastle - Ottawa Quality Assessment Scale Cohort Studies

Note: A study can be awarded a maximum of one star for each numbered item within the Selection and Outcome categories. A maximum of two stars can be given for Comparability

**Selection**
1) Representativeness of the exposed cohort
a) truly representative of the average **(i.e. ethnic minority asthma patients)** in the community ❋<br>b) somewhat representative of the average **(i.e. ethnic minority asthma patients)** in the community ❋<br>c) selected group of users e.g. nurses, volunteers<br>d) no description of the derivation of the cohort
2) Selection of the non exposed cohort
a) drawn from the same community as the exposed cohorts (i.e. **White / Caucasian / non-minority asthma patients)** ❋<br>b) drawn from a different source<br>c) no description of the derivation of the non exposed cohort
3) Ascertainment of exposure
a) secure record (e.g. **medical, insurance, health records)** ❋<br>b) structured interview ❋<br>c) written self report<br>d) no description
4) Demonstration that outcome of interest was not present at start of study
a) yes ❋<br>b) no
**Comparability**
1) Comparability of cohorts on the basis of the design or analysis
a) study controls for (**asthma severity**) ❋<br>b) study controls for any additional factor (**e.g. sex, age, socioeconomic status, comorbidity)** ❋
**Outcome**
1) Assessment of outcome
a) independent blind assessment (e.g. **health records**) ❋<br>b) record linkage (e.g. **health records**) ❋<br>c) self report (i.e. **no reference to original health records or documented source to confirm the outcome**)<br>d) no description
2) Was follow-up long enough for outcomes to occur
a) yes (**3 months and over)** ❋<br>b) no
3) Adequacy of follow up of cohorts
a) complete follow up - all subjects accounted for ❋<br>b) subjects lost to follow up unlikely to introduce bias - small number lost **(<5%)** follow up, (or description provided of those lost) ❋<br>c) follow up rate **< 80%** and no description of those lost

d) no statement

Threshold between good and poor-quality studies

Studies were rated poor-quality if:

- They did not adjust, by design or analysis, any factor
- They had less than 2 stars in the outcome section
- They had less than 3 stars in the selection section
- They had an overall rating of less than 6 stars

NOTE: The above quality assessment criteria were adapted for case-control and cross-sectional studies. However, regarding cross-sectional studies, studies were assigned a single star for ascertainment of the exposure, if they had used a validated tool, or described the measurement tool within the study or in their study protocol.

Table S5: Methodological quality assessment of the included studies using Newcastle-Ottawa Scale

Cohort Study

Study	Selection				Comparability	Outcomes			Quality Rating
	Representative-ness	Selection	Exposure Ascertainment	Outcome not present at start of study	Comparability	Outcome Assessment	Sufficient follow-up period	Adequacy of follow-up	
Mitchell 1988[1]	*	*	-	*	*	*	*	*	Good
Lozano 1995[2]	*	*	*	*	*	*	*	*	Good
Sarpong 1997[3]	*	*	-	*	*	*	*	*	Good
Joseph 1998[4]	*	*	*	*	*	*	*	*	Good
Blixen 1999[5]	*	*	*	*	*	*	*	*	Good
Eisner 2001[6]	*	*	-	*	*	-	*	-	Poor
Ortega 2001a[7]	*	*	-	*	-	-	*	*	Poor
Ortega 2001b[8]	*	*	-	*	-	-	*	*	Poor
Amre 2002[9]	*	*	*	*	*	-	*	*	Good
Diette 2002[10]	*	*	-	*	*	-	*	-	Poor
Lafata 2002[11]	*	*	*	*	*	*	*	*	Good
Weber 2002[12]	*	*	*	*	*	-	*	-	Poor
Bloomberg 2003[13]	*	*	*	*	-	*	*	*	Poor
Boudreaux 2003a[14]	*	*	-	*	-	*	*	*	Poor
Boudreaux 2003b[15]	*	*	-	*	*	-	*	-	Poor
Shields 2004[16]	*	*	-	*	*	*	*	*	Good
Carroll 2005[17]	*	*	*	*	*	*	*	*	Good
Griswold 2005[18]	*	*	-	*	*	*	*	*	Good
Ash 2006[19]	-	*	*	*	*	*	*	*	Good
Erickson 2007[20]	*	*	-	*	*	*	*	-	Good
Haselkorn 2008[21]	*	*	-	*	*	-	*	*	Good
Chandra 2009[22]	*	*	*	*	-	*	*	*	Poor
Haselkorn 2009[23]	*	*	-	*	*	-	*	*	Good
Carroll 2010[24]	*	*	*	*	-	*	-	*	Poor
Hasegawa 2014[25]	*	*	*	*	-	*	*	-	Poor

Kenyon 2014[26]	*	*	*	*	*	*	*	-	Good
Auger 2015[27]	*	*	*	*	*	*	*	-	Good
Venkat 2015[28]	*	*	*	*	-	*	-	*	Poor
Hull 2016[29]	*	*	-	*	*	*	*	*	Good
Mitchell 2016[30]	*	*	-	*	-	-	*	*	Poor
Franklin 2017[31]	*	*	-	*	-	*	*	*	Poor
Parikh 2017[32]	*	*	-	*	*	*	*	*	Good
Grunwell 2018[33]	*	*	-	*	*	-	*	*	Good
Trent 2018[34]	*	*	*	*	-	*	*	*	Poor
Aratani 2019[35]	*	*	*	*	*	*	*	*	Good
Fitzpatrick 2019[36]	*	*	-	*	-	*	*	*	Poor
Zein 2020[37]	*	*	*	*	-	*	*	*	Poor
Kraft 2021[38]	*	*	*	*	-	*	*	*	Poor
Sheikh 2021[39]	*	*	-	*	-	-	*	*	Poor
Adejare 2022[40]	*	*	*	*	-	*	*	*	Poor
Beuther 2022[41]	*	*	-	*	*	*	*	*	Good
Busby 2022[42]	*	*	*	*	*	*	*	*	Good
Lugogo 2022[43]	*	*	*	*	-	*	*	*	Poor
Redmond 2022[44]	*	*	*	*	*	*	*	*	Good

Cross-sectional Study

Study	Selection				Comparability	Exposures		Quality Rating
	Representative-ness	Sample size	Response rate	Exposure Ascertainment	Comparability	Outcome Assessment	Statistical test	
Zoratti 1998[45]	*	*	*	*	-	**	*	Poor
Meurer 2000[46]	*	*	-	*	*	*	*	Good
Krishnan 2001[47]	*	*	*	*	-	*	*	Poor
Grant 2005[48]	*	*	*	*	*	*	*	Good

Meng 2006[49]	*	*	-	*	*	*	*	Good
DeWalt 2007[50]	*	*	-	*	*	*	*	Good
Forester 2008[51]	*	*	*	*	-	*	*	Poor
Crocker 2009[52]	*	*	-	*	*	*	*	Good
Diette 2009[53]	*	*	-	*	*	*	*	Good
Gorman 2009[54]	*	*	-	*	-	*	-	Poor
Kim 2009[55]	*	*	-	*	*	*	*	Good
Wright 2009[56]	*	*	*	*	-	*	*	Poor
Canino 2012[57]	*	-	*	-	-	*	*	Poor
Lee 2014[58]	*	*	-	*	*	*	*	Good
Hughes 2017[59]	*	*	*	*	*	*	*	Good
Zhang 2017[60]	*	*	-	*	*	*	*	Good
Deshpande 2018[61]	*	*	-	*	*	*	*	Good
Cremer 2020[62]	*	*	-	*	*	*	*	Good
Urquhart 2020[63]	*	*	-	*	*	*	*	Good
Banta 2021[64]	*	*	*	*	*	*	*	Good

Case control Study

**Study**	Selection				Comparability	Outcomes			Quality Rating
	Case Definition	Representative-ness	Control Selection	Control Definition	Comparability	Exposure Ascertainment	Consistent between cases and controls	Non-Response rate	
Wells 2015[65]	-	*	-	*	-	*	*	-	Poor

Table S6: Adjustment factors reported from studies reporting asthma-related healthcare use by ethnicity

Study ID	Age	Sex	SES	Income	Insurance	Education	BMI	Asthma severity	Asthma drug	Smoking status	Comorbidity	Health status	Location	Literacy
Mitchell 1988[1]	×	×	✓	×	×	×	×	×	×	×	×	×	×	×
Lozano 1995[2]	✓	✓	×	×	×	×	×	×	×	×	×	×	✓	×
Sarpong 1997[3]	×	×	×	×	×	×	×	×	×	×	×	×	×	×
Joseph 1998[4]	×	×	×	×	×	×	×	×	×	×	×	×	×	×
Zoratti 1998[45]	×	×	×	×	×	×	×	×	×	×	×	×	×	×
Blixen 1999[5]	✓	✓	×	✓	×	×	×	×	×	×	×	×	×	×
Meurer 2000[46]	✓	✓	×	×	×	×	×	✓	×	×	×	×	×	×
Eisner 20011[6]	✓	✓	×	✓	×	✓	×	✓	×	✓	×	✓	×	×
Krishnan 2001[47]	×	×	×	×	×	×	×	×	×	×	×	×	×	×
Ortega 2001a[7]	×	×	×	×	×	×	×	×	×	×	×	×	×	×
Ortega 2001b[8]	×	×	×	×	×	×	×	×	×	×	×	×	×	×
Amre 2002[9]	✓	✓	×	×	×	×	×	×	✓	✓	×	×	×	×
Diette 2002[10]	✓	✓	×	×	×	✓	×	✓	×	✓	✓	✓	×	×
Lafata 2002[11]	✓	✓	×	✓	×	×	×	×	×	×	×	×	×	×
Weber 2002[12]	✓	✓	×	×	×	×	×	✓	✓	×	×	×	×	×
Bloomberg 2003[13]	✓	✓	×	×	✓	×	×	×	×	×	×	×	×	×
Boudreaux 2003a[14]	✓	✓	×	✓	✓	×	×	×	✓	×	×	×	×	×
Boudreaux 2003b[15]	×	×	×	×	×	×	×	×	×	×	×	×	×	×
Shields 2004[16]	✓	✓	×	×	✓	×	×	×	×	×	×	×	✓	×
Carroll 2005[17]	✓	×	×	×	×	✓	×	×	×	✓	×	×	✓	×
Grant 2005[48]	✓	✓	×	✓	×	×	×	×	✓	×	×	×	×	×
Griswold 2005[18]	✓	✓	×	×	✓	✓	×	×	✓	×	×	×	×	×
Ash 2006[19]	✓	✓	×	×	×	×	×	×	×	×	✓	×	×	×
Meng 2006[49]	✓	✓	×	✓	✓	✓	×	✓	×	✓	×	✓	✓	×
DeWalt 2007[50]	✓	×	×	✓	×	×	×	✓	✓	✓	×	×	×	✓
Erickson 2007[20]	×	×	×	×	×	×	×	×	×	×	×	×	×	×
Forester 2008[51]	✓	×	×	×	×	×	×	×	✓	×	×	×	×	✓
Haselkorn 2008[21]	×	×	×	×	×	×	×	×	×	×	×	×	×	×

Chandra 2009[22]	×	×	×	×	×	×	×	×	×	×	×	×	×	×
Crocker 2009[52]	✓	✓	×	✓	✓	×	✓	✓	×	✓	×	×	✓	×
Diette 2009[53]	✓	✓	×	✓	✓	✓	✓	×	×	×	×	×	×	×
Gorman 2009[54]	×	×	×	×	×	×	×	×	×	×	×	×	×	×
Haselkorn 2009[23]	✓	✓	×	✓	×	×	✓	×	✓	✓	×	×	×	×
Kim 2009[55]	✓	✓	×	×	×	×	×	×	×	×	×	×	✓	×
Wright 2009[56]	×	×	×	×	×	×	×	×	×	×	×	×	×	×
Carroll 2010[24]	×	×	×	×	×	×	×	×	×	×	×	×	×	×
Canino 2012[57]	×	×	×	×	×	×	×	×	×	×	×	×	×	×
Hasegawa 2014[25]	×	×	×	×	×	×	×	×	×	×	×	×	×	×
Kenyon 2014[26]	✓	✓	×	✓	×	×	×	×	✓	×	×	×	×	×
Lee 2014[58]	×	✓	×	×	✓	×	×	✓	✓	✓	×	✓	×	×
Auger 2015[27]	×	×	×	×	×	×	×	×	×	×	×	×	×	×
Venkat 2015[28]	×	×	×	×	×	×	×	×	×	×	×	×	×	×
Wells 2015[65]	×	×	×	×	×	×	×	×	×	×	×	×	×	×
Hull 2016[29]	✓	✓	✓	×	×	×	×	✓	✓	✓	✓	×	×	×
Mitchell 2016[30]	×	×	×	×	×	✓	×	×	×	×	×	×	×	×
Franklin 2017[31]	×	×	×	×	×	×	×	×	×	×	×	×	×	×
Hughes 2017[59]	✓	✓	×	✓	×	✓	×	×	×	×	×	×	✓	×
Parikh 2017[32]	✓	×	×	×	✓	×	×	✓	×	×	✓	×	×	×
Zhang 2017[60]	✓	✓	×	✓	✓	✓	×	×	×	✓	×	×	×	×
Deshpande 2018[61]	✓	✓	×	×	×	✓	×	×	✓	×	×	✓	×	×
Grunwell 2018[33]	✓	×	×	✓	×	×	×	✓	×	×	✓	×	×	×
Trent 2018[34]	×	×	×	×	×	×	×	×	×	×	×	×	×	×
Aratani 2019[35]	×	✓	×	×	×	×	×	×	×	×	×	×	×	×
Fitzpatrick 2019[36]	×	×	×	✓	×	✓	×	×	×	✓	×	×	×	×
Cremer 2020[62]	×	×	×	×	✓	×	×	×	✓	✓	✓	×	×	×
Urquhart 2020[63]	✓	✓	×	✓	✓	✓	×	×	×	×	×	✓	✓	×
Zein 2020[37]	×	×	×	×	×	×	×	×	×	×	×	×	×	×
Banta 2021[64]	✓	✓	×	×	×	×	✓	×	×	×	×	×	×	×
Kraft 2021[38]	×	×	×	×	×	×	×	×	×	×	×	×	×	×

Sheikh 2021[39]	×	×	×	×	×	×	×	×	×	×	×	×	×	×
Adejare 2022[40]	×	×	×	×	×	×	×	×	×	×	×	×	×	×
Beuther 2022[41]	×	×	×	×	×	×	×	×	×	×	×	×	×	×
Busby 2022[42]	✓	✓	×	×	×	×	×	✓	×	×	×	×	×	×
Lugogo 2022[43]	×	×	×	×	×	×	×	×	×	×	×	×	×	×
Redmond 2022[44]	✓	✓	×	×	×	×	×	✓	✓	×	×	×	✓	×

NOTE: SES, socioeconomic status; BMI, body mass index; Smoking; smoking status, exposure, environmental tobacco smoke; Location, urban or rural area of residence; √, Yes; ×, No

TABLE S7 - Subgroup Analyses: pooled effect on asthma-related healthcare utilisation by various factors

Subgroup	ED Visit				Hospitalisation				Hospital Readmission			
	N	OR (95% CI's)	I^2	*P*	N	OR (95% CI's)	I^2	*P*	N	OR (95% CI's)	I^2	*P*
**Race**				0.001				0.068				<0.001
Black	33	1.86 (1.68, 2.06)	90.6		30	1.69 (1.40, 2.06)	86.1		5	1.76 (1.61, 1.91)	65.4	
Hispanic	22	1.52 (1.35, 1.71)	89.6		13	1.33 (1.09, 1.62)	61.2					
Asian	5	0.97 (0.59, 1.61)	81.8		4	1.42 (0.85, 2.38)	88.8					
Other	8	1.13 (0.86, 1.48)	73.3		6	1.23 (0.88, 1.72)	30.8		4	1.05 (0.97, 1.13)	0.0	
**Age**				0.942				0.840				
Paediatric	18	1.72 (1.45, 2.04)	89.3		13	1.63 (1.37, 1.94)	36.9					
Other	18	1.79 (1.45, 2.18)	85.8		17	1.62 (1.44, 1.83)	49.1					
**Time period**				0.745								
Pre-2010	30	1.79 (1.54, 2.08)	90.7									
Post-2010	6	1.58 (1.29, 1.94)	74.2									

References:

1. Mitchell EA, Quested C. Why are Polynesian children admitted to hospital for asthma more frequently than European children? *N Z Med J*. Published online 1988.

2. Lozano P, Connell FA, Koepsell TD. Use of Health Services by African-American Children With Asthma on Medicaid. *JAMA J Am Med Assoc*. Published online 1995. doi:10.1001/jama.1995.03530060043031

3. Sarpong SB, Karrison T. Sensitization to indoor allergens and the risk for asthma hospitalization in children. *Ann Allergy, Asthma Immunol*. 1997;79(5):455-459. doi:10.1016/S1081-1206(10)63043-8

4. Joseph CLM, Havstad SL, Ownby DR, Johnson CC, Tilley BC. Racial differences in emergency department use persist despite allergist visits and prescriptions filled for antiinflammatory medications. *J Allergy Clin Immunol*. 1998;101(4 I):484-490. doi:10.1016/S0091-6749(98)70355-0

5. Blixen CE, Havstad S, Tilley BC, Zoratti E. A comparison of asthma-related healthcare use between African-Americans and Caucasians belonging to a health maintenance organization (HMO). *J Asthma*. Published online 1999. doi:10.3109/02770909909056317

6. Eisner MD, Katz PP, Yelin EH, Shiboski SC, Blanc PD. Risk factors for hospitalization among adults with asthma: The influence of sociodemographic factors and asthma severity. *Respir Res*. 2001;2(1):53-60. doi:10.1186/rr37

7. Ortega AN, Belanger KD, Paltiel AD, Horwitz SM, Bracken MB, Leaderer BP. Use of Health Services by Insurance Status among Children with Asthma. *Med Care*. 2001;39(10):1065-1074. doi:10.1097/00005650-200110000-00004

8. Ortega AN, Belanger KD, Bracken MB, Leaderer BP. A childhood asthma severity scale: Symptoms, medications, and health care visits. *Ann Allergy, Asthma Immunol*. 2001;86(4):405-413. doi:10.1016/S1081-1206(10)62486-6

9. Amre D, Infante-Rivard C, Gautrin D, Malo JL. Socioeconomic status and utilization of health care services among asthmatic children. *J Asthma*. 2002;39(7):625-631. doi:10.1081/JAS-120014927

10. Diette GB, Krishnan JA, Dominici F, et al. Asthma in older patients: Factors associated with hospitalization. *Arch Intern Med*. Published online 2002. doi:10.1001/archinte.162.10.1123

11. Lafata JE, Xi H, Divine G. Risk factors for emergency department use among children with asthma using primary care in a managed care environment. *Ambul Pediatr*. 2002;2(4):268-275. doi:10.1367/1539-4409(2002)002<0268:RFFEDU>2.0.CO;2

12. Weber EJ, Silverman RA, Callaham ML, et al. A prospective multicenter study of factors associated with hospital admission among adults with acute asthma. *Am J Med*. 2002;113(5):371-378. doi:10.1016/S0002-9343(02)01242-1

13. Bloomberg GR, Trinkaus KM, Fisher EB, Musick JR, Strunk RC. Hospital readmissions for childhood asthma: A 10-year metropolitan study. *Am J Respir Crit Care Med*.

2003;167(8):1068-1076. doi:10.1164/rccm.2201015

14. Boudreaux ED, Emond SD, Clark S, Cawargo CA. Acute asthma among adults presenting to the emergency department: The role of race/ethnicity and socioeconomic status. *Chest*. 2003;124(3):803-812. doi:10.1378/chest.124.3.803

15. Boudreaux ED, Emond SD, Clark S, Camargo CA. Race/Ethnicity and Asthma Among Children Presenting to the Emergency: Differences in Disease Severity and Management. 2012;111(2020).

16. Shields AE, Comstock C, Weiss KB. Variations in Asthma Care by Race/Ethnicity Among Children Enrolled in a State Medicaid Program. 2015;113(3).

17. Carroll KN, Griffin MR, Gebretsadik T, Shintani A, Mitchel E, Hartert T V. Racial differences in asthma morbidity during pregnancy. *Obstet Gynecol*. Published online 2005. doi:10.1097/01.AOG.0000164471.87157.4c

18. Griswold SK, Nordstrom CR, Clark S, Gaeta TJ, Price ML, Camargo CA. Asthma exacerbations in North American adults: Who are the "frequent fliers" in the emergency department? *Chest*. Published online 2005. doi:10.1378/chest.127.5.1579

19. Ash M, Brandt S. Disparities in asthma hospitalization in Massachusetts. *Am J Public Health*. 2006;96(2):358-362. doi:10.2105/AJPH.2004.050203

20. Erickson SE, Iribarren C, Tolstykh I V., Blanc PD, Eisner MD. Effect of race on asthma management and outcomes in a large, integrated managed care organization. *Arch Intern Med*. 2007;167(17):1846-1852. doi:10.1001/archinte.167.17.1846

21. Haselkorn T, Lee JH, Mink DR, Weiss ST. Racial disparities in asthma-related health outcomes in severe or difficult-to-treat asthma. *Ann Allergy, Asthma Immunol*. 2008;101(3):256-263. doi:10.1016/S1081-1206(10)60490-5

22. Chandra D, Clark S, Camargo CA. Race/ethnicity differences in the inpatient management of acute asthma in the United States. *Chest*. Published online 2009. doi:10.1378/chest.08-1812

23. Haselkorn T, Zeiger RS, Chipps BE, et al. Recent asthma exacerbations predict future exacerbations in children with severe or difficult-to-treat asthma. *J Allergy Clin Immunol*. 2009;124(5):921-927. doi:10.1016/j.jaci.2009.09.006

24. Carroll CL, Uygungil B, Zucker AR, Schramm CM. Identifying an at-risk population of children with recurrent near-fatal asthma exacerbations. *J Asthma*. 2010;47(4):460-464. doi:10.3109/02770903.2010.481344

25. Hasegawa K, Tsugawa Y, Brown DFM, Camargo CA. A population-based study of adults who frequently visit the emergency department for acute asthma: California and Florida, 2009-2010. *Ann Am Thorac Soc*. 2014;11(2):158-166. doi:10.1513/AnnalsATS.201306-166OC

26. Kenyon CC, Melvin PR, Chiang VW, Elliott MN, Schuster MA, Berry JG. Rehospitalization for childhood asthma: Timing, variation, and opportunities for intervention. *J Pediatr*. 2014;164(2):300-305. doi:10.1016/j.jpeds.2013.10.003

27. Auger KA, Kahn RS, Davis MM, Simmons JM. Pediatric asthma readmission: Asthma

knowledge is not enough? *J Pediatr*. 2015;166(1):101-108.e1. doi:10.1016/j.jpeds.2014.07.046

28. Venkat A, Hasegawa K, Basior JM, et al. Race/ethnicity and asthma management among adults presenting to the emergency department. *Respirology*. 2015;20(6):994-997. doi:10.1111/resp.12572

29. Hull SA, McKibben S, Homer K, Taylor SJ, Pike K, Griffiths C. Asthma prescribing, ethnicity and risk of hospital admission: An analysis of 35,864 linked primary and secondary care records in East London. *npj Prim Care Respir Med*. 2016;26(March). doi:10.1038/npjpcrm.2016.49

30. Mitchell SJ, Bilderback AL, Okelo SO. Racial Disparities in Asthma Morbidity among Pediatric Patients Seeking Asthma Specialist Care. *Acad Pediatr*. 2016;16(1):64-67. doi:10.1016/j.acap.2015.06.010

31. Franklin JM, Grunwell JR, Bruce AC, Smith RC, Fitzpatrick AM. Predictors of emergency department use in children with persistent asthma in metropolitan Atlanta, Georgia. *Ann Allergy, Asthma Immunol*. Published online 2017. doi:10.1016/j.anai.2017.04.008

32. Parikh K, Berry J, Hall M, et al. Racial and Ethnic Differences in Pediatric Readmissions for Common Chronic Conditions. *J Pediatr*. 2017;186:158-164.e1. doi:10.1016/j.jpeds.2017.03.046

33. Grunwell JR, Travers C, Fitzpatrick AM. Inflammatory and comorbid features of children admitted to a PICU for status asthmaticus. *Pediatr Crit Care Med*. 2018;19(11):E585-E594. doi:10.1097/PCC.0000000000001695

34. Trent SA, Hasegawa K, Ramratnam SK, Bittner JC, Camargo CA. Variation in asthma care at hospital discharge by race/ethnicity groups. *J Asthma*. Published online 2018. doi:10.1080/02770903.2017.1378356

35. Aratani Y, Nguyen HA, Sharma V. Asthma-related emergency department visits among low-income families with young children by race/ethnicity and primary language. *Pediatr Emerg Care*. 2020;36(11):e636-e640. doi:10.1097/PEC.0000000000001430

36. Fitzpatrick AM, Gillespie SE, Mauger DT, et al. Racial disparities in asthma-related health care use in the National Heart, Lung, and Blood Institute's Severe Asthma Research Program. *J Allergy Clin Immunol*. Published online 2019. doi:10.1016/j.jaci.2018.11.022

37. Zein JG, Wu CP, Attaway AH, Zhang P, Nazha A. Novel Machine Learning Can Predict Acute Asthma Exacerbation. *Chest*. 2021;159(5):1747-1757. doi:10.1016/j.chest.2020.12.051

38. Kraft M, Brusselle G, FitzGerald JM, et al. Patient characteristics, biomarkers and exacerbation risk in severe, uncontrolled asthma. *Eur Respir J*. 2021;58(6). doi:10.1183/13993003.00413-2021

39. Sheikh SI, Ryan-Wenger NA, Pitts J, Britt R, Paul G, Ulrich L. Impact of guideline adherence and race on asthma control in children. *World J Pediatr*. 2021;17(5):500-507. doi:10.1007/s12519-021-00458-5

40. Adejare AA, Gautam Y, Madzia J, Mersha TB. Unraveling racial disparities in asthma emergency department visits using electronic healthcare records and machine learning. *J Asthma*. 2022;59(1):79-93. doi:10.1080/02770903.2020.1838539

41. Beuther DA, Murphy KR, Zeiger RS, et al. Asthma Impairment and Risk Questionnaire Control Level Predicts Future Risk of Asthma Exacerbations. *J Allergy Clin Immunol Pract*. Published online 2022. doi:10.1016/j.jaip.2022.08.017

42. Busby J, Heaney LG, Brown T, et al. Ethnic Differences in Severe Asthma Clinical Care and Outcomes: An Analysis of United Kingdom Primary and Specialist Care. *J Allergy Clin Immunol Pract*. 2022;10(2):495-505.e2. doi:10.1016/j.jaip.2021.09.034

43. Lugogo N, Judson E, Haight E, et al. Severe asthma exacerbation rates are increased among female, Black, Hispanic, and younger adult patients: results from the US CHRONICLE study. *J Asthma*. 2021;59(12):2495-2508. doi:10.1080/02770903.2021.2018701

44. Redmond C, Heaney LG, Chaudhuri R, et al. Benefits of specialist severe asthma management: demographic and geographic disparities. *Eur Respir J*. Published online 2022:2200660. doi:10.1183/13993003.00660-2022

45. Zoratti EM, Havstad S, Rodriguez J, Robens-Paradise Y, Lafata JE, McCarthy B. Health service use by African Americans and Caucasians with asthma in a managed care setting. *Am J Respir Crit Care Med*. 1998;158(2):371-377. doi:10.1164/ajrccm.158.2.9608039

46. Meurer JR, George V, Subichin SJ, et al. Risk factors for pediatric asthma emergency visits. *J Asthma*. Published online 2000. doi:10.3109/02770900009087303

47. Krishnan JA, Diette GB, Skinner EA, Clark BD, Steinwachs D, Wu AW. Race and sex differences in consistency of care with National Asthma Guidelines in managed care organizations. *Arch Intern Med*. 2001;161(13):1660-1668. doi:10.1001/archinte.161.13.1660

48. Grant EN, Malone A, Lyttle CS, Weiss KB. Asthma morbidity and treatment in the Chicago metropolitan area: One decade after national guidelines. *Ann Allergy, Asthma Immunol*. 2005;95(1):19-25. doi:10.1016/S1081-1206(10)61183-0

49. Meng YY, Babey SH, Brown ER, Malcolm E, Chawla N, Lim YW. Emergency department visits for asthma: The role of frequent symptoms and delay in care. *Ann Allergy, Asthma Immunol*. 2006;96(2):291-297. doi:10.1016/S1081-1206(10)61238-0

50. DeWalt DA, Dilling MH, Rosenthal MS, Pignone MP. Low Parental Literacy Is Associated With Worse Asthma Care Measures in Children. *Ambul Pediatr*. Published online 2007. doi:10.1016/j.ambp.2006.10.001

51. Forester JP, Ong BA, Fallot A. Can equal access to care eliminate racial disparities in pediatric asthma outcomes? *J Asthma*. Published online 2008. doi:10.1080/02770900801890448

52. Crocker D, Brown C, Moolenaar R, et al. Racial and ethnic disparities in asthma medication usage and health-care utilization: Data from the National Asthma Survey. *Chest*. Published online 2009. doi:10.1378/chest.09-0013

53. Diette GB, Sajjan S, Skinner EA, Weiss TW, Wu AW, Markson LE. Using the pediatric

asthma therapy assessment questionnaire to measure asthma control and healthcare utilization in children. *Patient*. 2009;2(4):233-241. doi:10.2165/11313820-000000000-00000

54. Gorman BK, Chu M. Racial and ethnic differences in adult asthma prevalence, problems, and medical care. *Ethn Heal*. 2009;14(5):527-552. doi:10.1080/13557850902954195

55. Kim H, Kieckhefer GM, Greek AA, Joesch JM, Baydar N. Health care utilization by children with asthma. *Prev Chronic Dis*. 2009;6(1).

56. Wright K. Disparities and predictors of emergency department use among California's African American, Latino, and white children, aged 1-11 years, with asthma. *Ethn Dis*. 2009;19(1):71-77.

57. Canino G, Garro A, Alvarez MM, et al. Factors associated with disparities in emergency department use among Latino children with asthma. *Ann Allergy, Asthma Immunol*. 2012;108(4):266-270. doi:10.1016/j.anai.2012.02.002

58. Lee JA, Reed PL, Berg JP. Asthma characteristics among older adults: Using the California health interview survey to examine asthma incidence, morbidity and ethnic differences. *J Asthma*. 2014;51(4):399-404. doi:10.3109/02770903.2013.879879

59. Hughes HK, Matsui EC, Tschudy MM, Pollack CE, Keet CA. Pediatric Asthma Health Disparities: Race, Hardship, Housing, and Asthma in a National Survey. *Acad Pediatr*. 2017;17(2):127-134. doi:10.1016/j.acap.2016.11.011

60. Zhang Q, Lamichhane R, Diggs LA. Disparities in emergency department visits in American children with asthma: 2006–2010. *J Asthma*. 2017;54(7):679-686. doi:10.1080/02770903.2016.1263315

61. Deshpande M, Look KA. Exploring factors associated with asthma-related emergency department visits among adults: A path analysis approach. *Res Soc Adm Pharm*. 2018;14(1):46-52. doi:10.1016/j.sapharm.2016.12.011

62. Cremer NM, Baptist AP. Race and Asthma Outcomes in Older Adults: Results from the National Asthma Survey. *J Allergy Clin Immunol Pract*. 2020;8(4):1294-1301.e7. doi:10.1016/j.jaip.2019.12.014

63. Urquhart A, Clarke P. US racial/ethnic disparities in childhood asthma emergent health care use: National Health Interview Survey, 2013–2015. *J Asthma*. Published online 2020. doi:10.1080/02770903.2019.1590588

64. Banta JE, Ramadan M, Alhusseini N, Aloraini K, Modeste N. Socio-demographics and asthma prevalence, management, and outcomes among children 1–11 years of age in California. *Glob Heal Res Policy*. 2021;6(1). doi:10.1186/s41256-021-00199-y

65. Wells RE, Garb J, Fitzgerald J, Kleppel R, Rothberg MB. Factors associated with emergency department visits in asthma exacerbation. *South Med J*. Published online 2015. doi:10.14423/SMJ.0000000000000275
